# Plasmon-enhanced Electrically Light-emitting from ZnO Nanorod Arrays/p-GaN Heterostructure Devices

**DOI:** 10.1038/srep25645

**Published:** 2016-05-16

**Authors:** Junfeng Lu, Zengliang Shi, Yueyue Wang, Yi Lin, Qiuxiang Zhu, Zhengshan Tian, Jun Dai, Shufeng Wang, Chunxiang Xu

**Affiliations:** 1 State Key Laboratory of Bioelectronics, School of Biological Science & Medical Engineering, Southeast University, Nanjing 210096, China; 2 Institute of Modern Optics & State Key Laboratory for Mesoscopic Physics, School of Physics,Peking University, Beijing 100871, China

## Abstract

Effective and bright light-emitting-diodes (LEDs) have attracted broad interests in fundamental research and industrial application, especially on short wavelength LEDs. In this paper, a well aligned ZnO nanorod arrays grown on the p-GaN substrate to form a heterostructured light-emitting diode and Al nanoparticles (NPs) were decorated to improve the electroluminescence performance. More than 30-folds enhancement of the electroluminescence intensity was obtained compared with the device without Al NPs decoration. The investigation on the stable and transient photoluminescence spectraof the ZnO nanorod arrays before and after Al NPs decoration demonstrated that the metal surface plasmon resonance coupling with excitons of ZnO leads to the enhancement of the internal quantum efficiency (IQE). Our results provide aneffective approach to design novel optoelectronic devices such as light-emitting diodes and plasmonic nanolasers.

Zinc oxide (ZnO), as a competent material with anintrinsic direct wide energy bandgap and largeexciton binding energy, has attracted considerable interest for short-wavelength photonic application in display, illumination, optical communication and information storage and biomedical detection^1^,^2^,^3^,^4^,^5^,^6^,^7^,^8^. Many reports have demonstrated its efficient ultravioletexcitonic emission in powders, thin films, light-emittingdiode (LED) and laser diodes under the different optical and electrical excitation^9^,^10^,^11^,^12^,^13^,^14^. However, the stable and reproducible p-type ZnO is still in challenge. Alternatively, an n-ZnO/p-GaN heterojunction has been suggested as an important candidate approach for device fabrication and application^15^,^16^, since GaN has similar fundamental bandgap energy of 3.4 eV, the same wurtzite crystal structure, and a low lattice constant mismatch of 1.9%. Especially, single crystalline one-dimensional ZnO nanostructures grown on GaN filmcan construct nano-sized heterojunctions with high carrier injection rate and nice electroluminescence performance due tothe high crystal quality and high surface-to-volume ratios^17^. Even so, some physical effect or technological approach are still required to further improve the IQE and the light extraction outward, which is very important to develop novel nanodevices and understand the indwelling scientificfoundation.

Recently, surface plasmon (SP), as collective charge oscillationat metal/dielectric interface, has been actively employed to improve the luminous efficiency of light-emitting materials and devices^18^,^19^,^20^,^21^,^22^. For example, in 2004, Okamoto *et al.*^23^ reporteda 14-fold PL enhancement of SP-coupled InGaN/GaN quantum well (QW) by usingsilver (Ag) layers toimprove theinternal quantum efficiencies. In 2008, Cheng *et al.*^24^ achieved 3-fold enhancement of the near band emission (NBE) of ZnO films through coupling with localized surface plasmons (LSPs) of the sputtered Ag islands. In addition, the metal Au is also used to enhance the PL of ZnO. In 2010, Cheng *et al.*^25^ observed 6-fold PL enhancement of NBE from ZnO nanorods by Au NPs decoration, while the defect-related emission was completelysuppressed. Next year, Niu *et al.*^26^ obtained 11-fold PL enhancement of NBE of Au/ZnO nanobelts compared with that of the bare one. In the case of electroluminescence, Kwon *et al.*^27^ inserted Ag NPsamong the multiple quantum well (MQW) interlayers to improve the emission efficiency of the blue LED through QW-SP coupling. Based on ZnO materials, Zhang *et al.*^28^ reported 2.5-fold enhancement of EL from ZnO-based heterojunction LEDs decorated with Ag NPs, and the improvement was attributed to the resonant coupling between excitons of ZnO and localized SPs in Ag NPs. Next year, they obtained 3.7-fold enhancement of EL by optimizing Ag localized SPs^29^. The same year, Liu *et al.*^30^ achieved 7-fold EL enhancement from Ag NPs decorated ZnO-based heterojunction LEDs via optimizing the thickness of MgO spacer layer. Recently, 13-folds enhancement of the UV LEDs has been obtained by the same group^31^ through embedding a ZnO nanorod array/p-GaN film heterostructure into an Ag-nanoparticles/PMMAcomposite. Further, a recent study by Qiao *et al.* demonstrates that the lasing threshold of aZnO laser diode can be effectively reduced by introducing Ag NP LSPs^32^. Many researches demonstratedthat the SP coupling at metal/semiconductor interface could increase the density of states, accelerate the recombination rate, and then enhance the internal quantum efficiency. However, thoseplasmon response spectra in the visible range presentthe big frequency mismatchfrom the NBE of ZnO, and hence suppress the effectively coupling between the metal SPs and the ZnO excitons. On the other hand, noble metals are always costly, which definitelyrestricts the potential for commercialization.

In view of the low cost and its abundance in the world, aluminium (Al) element has now been an important candidate of plasmonic material due to its negative real part and relatively low imaginary part of the dielectric function even at wavelength smaller than 200 nm^33^. ThereforeAl is a betterplasmonic material than either Au or Ag in the blueand UV range. More importantly, more than 170-fold PL enhancement of ZnO/Al hybrid microcavity induced by the resonant coupling between excitons of ZnO and SPs of Al nanoparticles (Al NPs) had been proved in our previous work^34^. In this paper, Al NP-decorated n-ZnO nanorods/p-GaN light-emitting array has been designed andconstructed, and great EL enhancement more than 30-fold was obtained compared with that of the bare one. The SP-coupling mechanism wasinvestigated systematically based on the stable and transient photoluminescence (PL) spectraof the bare and Al-decorated LEDs.

## Results and Discussion

**Figure 1:**
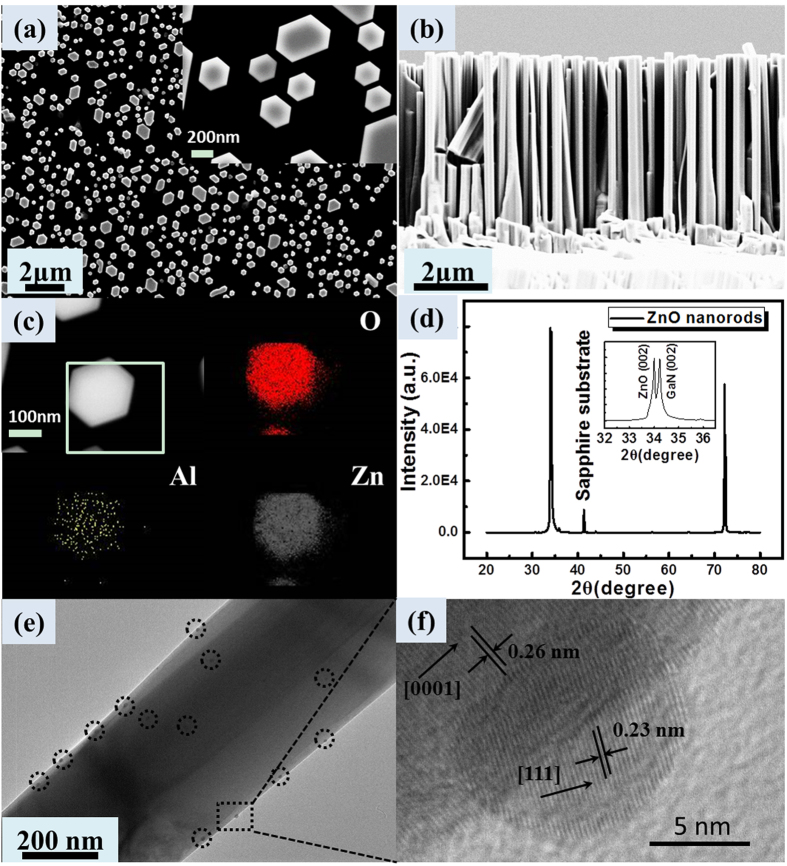
(**a**) Top and (**b**) side view SEM images of the as-grown ZnO nanorods. (**c**) Zn, O, Al element mapping images for the ZnO nanorod. (**d**) X-ray diffraction patterns of the ZnO nanorod arrays inserted with an enlarged view of the ZnO (002)/GaN (002) region. (**e**) TEM and (**f**) HRTEM images of ZnO nanorod decorated with Al nanoparticles.

Figure 1a,b are the top view and side view SEM images of the as-grown ZnO nanorod arrays. The diameter of the ZnO nanorods is ~300 nm from the enlarged top view SEM image inserted in Fig. 1a. The length of the ZnO nanorods is ~8 μm. For the fabrication of EL device, vertically well-aligned n-type ZnO nanorod arrays were grown on p-GaN (0001) substrate with the similar length by a simple vapor-phase transport process. Typical XRD pattern for the ZnO nanorods are shown in Fig. 1d. The high intensity of diffraction peaks reveals the growth direction along the [0001] direction in Fig. 1d. The two diffraction peaks at 34.46° and 72.59° are peculiar to (0002) and (0004) planes of the wurtzite ZnO. The strongest diffraction peak corresponds to the (0002) diffraction plane means that the nanorods mainly grow along the preferred [0001] direction, which was further verified by HRTEM shown in Fig. 1f. On the enlarged views of Fig. 1d’s inset, one can observe the ZnO (0002) diffraction peak on the left side of the GaN (0002) reflection. The patterns are typical of a perfectly textured ZnO material. The full width at half-maximum (FWHM) of the (0002) peak for ZnO and GaN are low and similar at 0.085° and 0.095°, respectively. The values are typical of a high-quality heterojunction. The elemental mapping profiles of the Al-decorated ZnO nanorod arrays confirm the existence of Al element, as shown in Fig. 1c. The elemental mapping images collected from the rectangle region in Fig. 1c reveals that the Zn element and O element distribute uniformly corresponding distinctly to the profile of the ZnO nanorod, while the Al element disperses on the surface of ZnO nanorod. Figure 1e,f show TEM and HRTEM images of ZnO nanorod decorated with Al NPs. It further confirms that the diameter of Al NPs is in the range of 15–20 nm. The HRTEM image reveals that the interplanar distance of 0.23 nm corresponds to the d spacing of (111) lattice plane of Al zinc blende structure.

**Figure 2:**
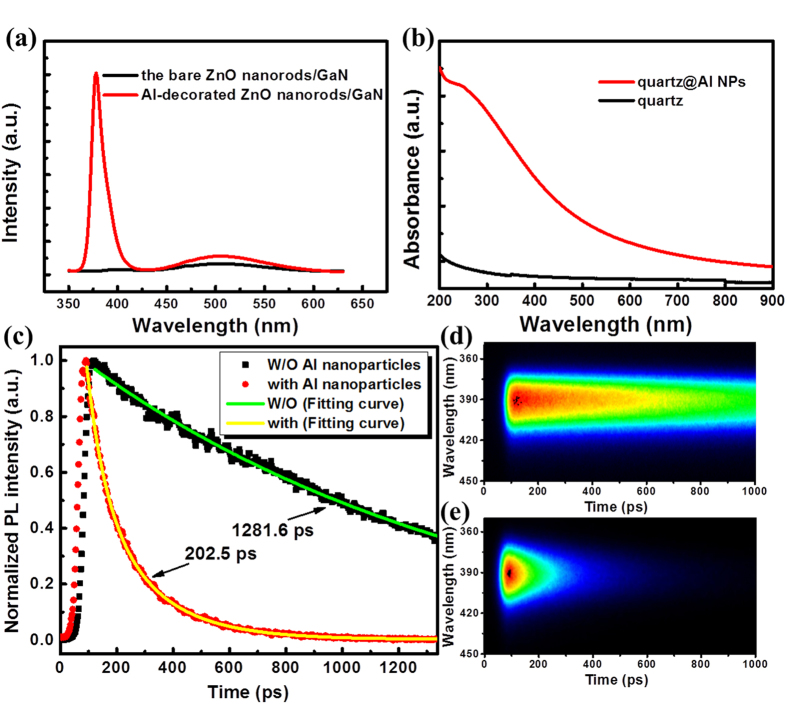
(**a**) PL spectraof the ZnO nanorodsbefore and after Al NPs decoration. (**b**) Transmittance spectra of quartz substrate without and with Al NPs under the same sputtering conditions as the Al-decorated sample.(**c**) The normalized TRPL spectra and exponential decay fitting curve at the wavelength of 390 nm. Temporal spectroscopic profile of (**d**) the bare and (**e**) Al-decorated ZnO nanorods excited by 295 nm laser and collected by a streak camera.

To examine optical properties of the as-grown ZnO nanorod arrays before and after decoration of Al NPs, the PL spectra of these two samples and the absorption spectra of quartz substrate with and without Al NPs measurements have been performed at room temperature, as shown in Fig. 2a,b. Before Al NPs decoration, a weak ultraviolet emission assigned to the NBE of ZnO and a strong green emission related with the defect in ZnO can be observed. After decoration with Al NPs, the intensity of the ultraviolet emission increased obviously, while the defect-related emission was suppressed. More than 40-fold enhancement was obtained compared with that of the bare one, which was attributed to the direct resonant coupling between the excitons of ZnO and surface plasmons (SPs) of Al NPs as mentioned before. The absorption spectra also verify that PLenhancementresults from the energy couplingbetween SPs and excitons. To gain more insights of the coupling mechanism, time-resolved photoluminescence (TRPL) measurements were performed at room temperature shown in Fig. 2a. The spontaneous emission decay rate of ZnO nanorods is significantly enhanced in the plasmon-coupled device due to Purcell effect. Panels d and e in Fig. 2 show the temporal spectroscopic profile of the bare and the Al-decorated ZnO nanorods. All the normalized TRPL decays can be fitted using a monoexponential function well. The instrument temporal response has been deconvolved from the fits. The fitted decay times are ~202.5 ps for the Al-decorated sample, whereas ~1281.6 ps for the bare ZnO nanorods. The Purcell enhancement factor *F*_p_ quantifies the increase in spontaneous emission rate into a mode of interest, and can be derived from the measured TR-PL decay time as follows^25^:


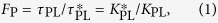



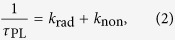



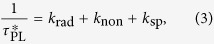


where *K*_PL_ and 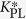 are the original and enhanced PL decay rate, respectively. *k*_rad_, *k*_non_ and *k*_sp_ are the radiative, non-radiative and SP-coupling recombination rate of electron-hole pairs, respectively. Using *τ*_PL_ = 1281.6 ps and 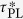 = 202.5 ps, an approximate 6.3 time enhancement of the spontaneous emission rate is calculated for the Al-decorated sample. Based on the above results, the efficient resonant coupling has occurred between the SPs of Al nanoparticles and excitons of ZnO nanorods.

**Figure 3:**
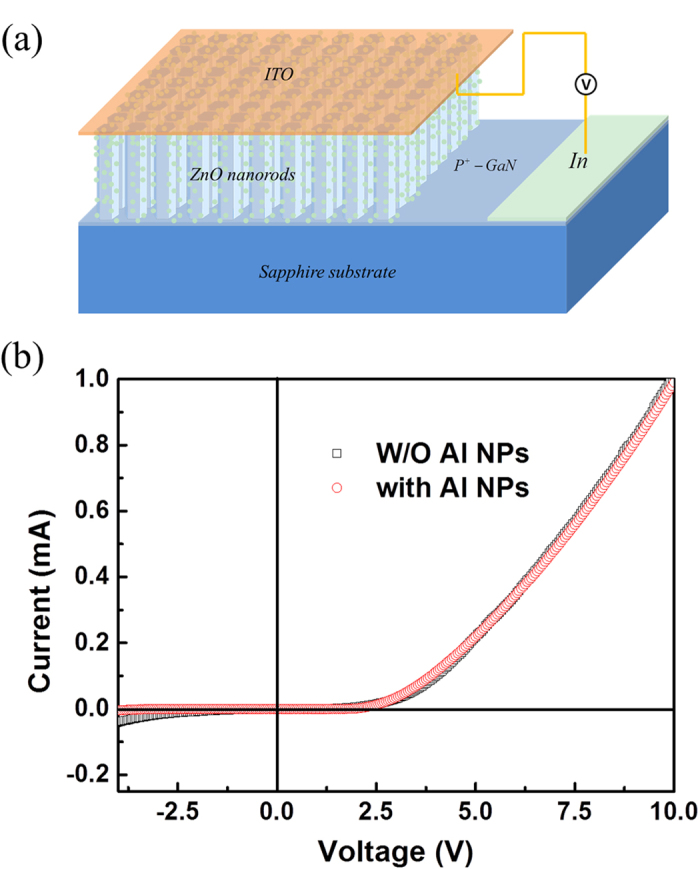
(**a**) The schematic diagram and (**b**) I-V characteristic of the device.

The ZnO nanorod array/p-GaN heterostructure was used to construct a light-emitting diode device as illustrated in the schematic of Fig. 3a. The top of the ZnO nanorods was contacted directly with an indium tin oxide (ITO) layer deposited on a transparent glass sheet. The GaN layer was contacted with an indium (In) electrode. Figure 3b shows the current versus voltage (I-V) characteristics for the same device before and after Al NPs decoration, which illustrates that the heterojunction behaves like an efficient diode with a current increasing rapidly under forward bias and blocking the current flow under reverse bias. The forward bias turn-on voltages of this device with and without Al nanoparticle decoration were 3.00 V and 3.10 V, respectively. These results indicate that decorating Al nanoparticles on the surface of ZnO nanorods has not caused the electrical properties to deteriorate.

**Figure 4:**
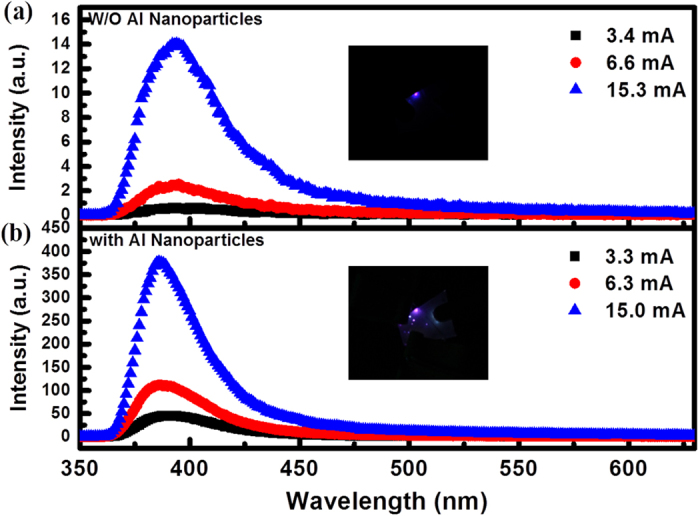
The EL spectra of the as-fabricated LED (**a**) without (**b**) with Al NPs decoration under different forward biases. The insets show two photographs of the EL emission from the ZnO nanorods/GaN heterojunction diodes (**a**) before and (**b**) after decorating with Al NPs.

Figure 4 shows the room temperature electroluminescence (EL) emission spectra of the bare and Al-decorated devices at different forward biases, inset with an image of the EL spots at the voltage of 30 V. For the bare device, EL spectra show a typical emission peak centered at 393 nm and extend unsymmetrically to 450 nm. However, the EL spectra of the Al-decorated device exhibit a slightly blue shift to 386 nm compared with that of the bare one. Meanwhile, more than 30-folds enhancement of the EL intensity from the Al-decorated device has been observed under the same forward bias. This indicates more efficient recombination of the excited carriers in the Al-decorated device under the electrically driven condition. Corresponding to the enhancement of the PL spectra, the efficient resonant coupling occurred between excitons of ZnO and SPs of Al NPs under electrically driven. The spontaneous recombination rate are increased shown in the transient PL spectra, so are the internal quantum efficiency (*η*_int_), which is given by the ratio of *k*_rad_ and *k*_non_^23^,


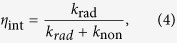


While the enhanced efficiencies 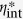 can be related to the coupling rate *k*_sp_ by the relationship,


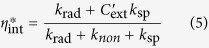


where 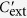 is the probability of photon extraction from the SP’s energy, and is decided by the ratio of the light scattering and dumping of electron vibration. It can be seen that the *η*_int_^*^ increased obviously due to the introduction of the SPs coupling rate, and also suppress the nonradiative transition. Combining equations (1, 2, 3, 4, 5), the IQE ratio between two samples can be obtained as follows:


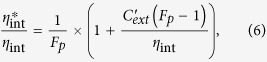


**Figure 5:**
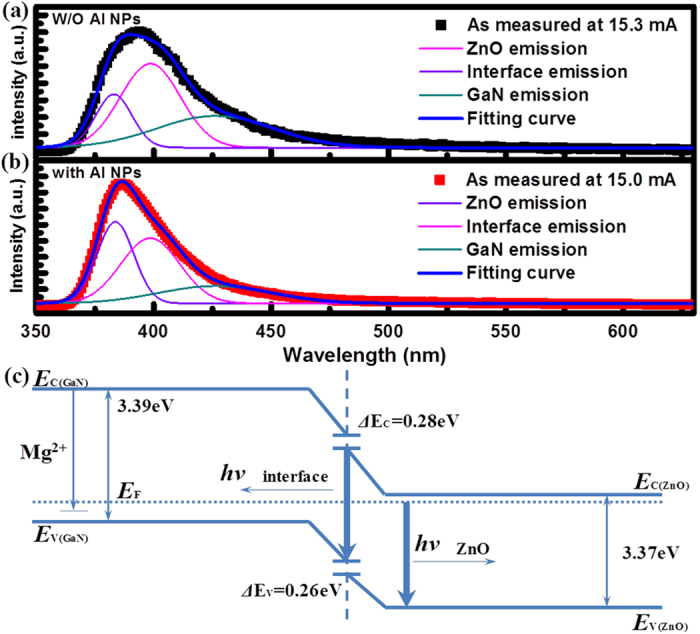
Gaussian deconvoluted three distinct sub-bands of a representative EL spectrum of ZnO nanorods/GaN heterojunction LED. (**a**) before and (**b**) after Al NPs decoration. (**c**)The energy band structure of the as-fabricated LED and the corresponding recombination.

In order to further catch at the recombination mechanism, a multipeak Guassian fit gives three Guassian bands, centering at ~385 nm, ~400 nm, and ~425 nm for the both bare and Al-decorated devices, respectively. As shown in Fig. 5(a,b), the Guassian curve fits well with the experimental curve. Compared with the PL spectra of the two devices, the UV and blue emission bands centered at 385 nm and 425 nm, respectively, is attributed to the NBE recombinationin ZnO and the electron transition from the conductionband to the deep Mg acceptor level in the Mg-doped p-GaN film, while the emission band centeredat 400 nm results from the interface recombination of the electrons in n-ZnO andholes in p-GaN due to the formation of the energybarrier at the ZnO/GaN heterostructural interface, then the barrier heights at the interface for theholes and the electrons are 0.26 eV and 0.28 eV, respectively. The band diagram of the proposed mechanism for therecombination processes considering the interfacialbarriers and the band diagram was obtained, as shown in Fig. 5c. In addition, the ratio of the emission centered at 400 nm induced by interface recombination to the ultraviolet emission from ZnO nanorods increased with the decoration of Al nanoparticles, which revealed that the efficient coupling occurs between the excitons of ZnO and the SPs of Al. Figure 2b shows that the absorption of Al NPs mainly exists in UV region, which is more benefit for the recombination of ZnO excitons. In the view of the Gaussian fitting curve in Fig. 5b, the enhanced ZnO UV emission and slightly blue shift were due to the higher SP coupling in the shorter wavelength. The SP enhancement of n-ZnO/p-GaN EL provides a promising method for developing highly efficient solid-state light sources.

In conclusion, we present SP-enhanced ZnO nanorod arrays/p-GaN heterostructurallight-emitting diodes containing decoratedwith Al nanoparticles. In comparison with the bare UV LEDs, the enhancement of luminescence intensity with dozens of times can be observed in both PL and EL spectra from the Al-decorated one. TRPL results showedthat the PL decay time of LEDs decorated with Al nanoparticleswas significantly decreased compared to that of the bare LEDs. And the Purcell enhancement factor *F*_*p*_ reaches up to 6.3, which indicated that the spontaneousemission rate was increased by the energyresonant coupling betweenthe excitons of ZnO and the SPs of Alnanoparticles. These findings demonstrate that SP coupling is oneof the most interesting methods for developing efficient LEDs, as themetal can be used not only as an electrical contact but also for excitingplasmons.

## Methods

### Synthesis of ZnO nanorod arrays

ZnO nanorods were fabricated bya vapor phase transport method. Firstly, a mixture of high purity ZnOand graphite powders (1:1 in mass ratio) was placed in a quartz boat asthe source material. Then, the quartz boat was put in the sealed end of aquartz test tube 30 mm in diameter and 300 mm in length, while a cleaned Mg-doped GaN substrate was put in the open end of the tube. Finally, the whole test tube was transferred into a tube furnace, which had been previously heated to 1050 °C. Argon and oxygen (150:15 sccm) were introduced intothe furnace as the carrier gases. The reaction lasted 15 min. The Al nanoparticles were sputtered onto the ZnO nanorods by a radio frequency magnetic sputtering system. The chamber pressure was fixed at 2.0 Pa, the Ar flow was 50 sccm and the sputtering power was 100 W. The sputtering time lasted 3 min.

### LED Fabrication

The Mg-doped GaN with carrier concentration of 3.0 × 10^17^ cm^−3^ was used as p-type substrate for LED construction. As shown in the schematic diagram of the LED in Fig. 5(a), the ZnO nanorodswere grown on the p-GaN substrate,and a piece of ITO-coated glass was impacted on them. Theindium electrode was deposited on GaN using the electron beamevaporation system.

### Materials and devices characterization

The morphology and structure of the as-synthesized products were characterized by field emission scanning electron microscopy (FESEM, Carl Zeiss Ultra Plus) equipped with an X-ray energy dispersive spectrometer (EDS) (Oxford X-Max 50), X-ray diffraction (XRD-7000, Shimadzu) using Cu Kα radiation (λ = 0.15406 nm) and high-resolution transmissionelectron microscope (HRTEM, JEM-2100). The PLspectrum was measured by a fluorescence spectrophotometer (F-4600, Hitachi) with a Xe lamp at 325 nm as the excitation source. Time-resolved photoluminescence (TRPL) experiments were performed by an optically triggered streak camera system(C10910, Hamamatsu) at 295 nm from frequency doubling of the fundamental 35 fs pulses at 590 nm with a repetition rate of 1 KHz (OperA Solo, Coherent). The I-V characteristics and electrical properties were measured by Keithley 4200.

## Additional Information

**How to cite this article**: Lu, J. *et al.* Plasmon-enhanced Electrically Light-emitting from ZnO Nanorod Arrays/p-GaN Heterostructure Devices. *Sci. Rep.* **6**, 25645; doi: 10.1038/srep25645 (2016).

## References

[b1] Kinoshita A., Hirayama H., Ainoya M., Aoyagi Y. & Hirata A. Room-temperature Operation at 333 nm of Al_0.03_Ga_0.97_N/Al_0.25_Ga_0.75_N Quantum-well Light-emitting Diodes with Mg-doped Superlattice Layers. Appl. Phys. Lett. 77, 175–177 (2000).

[b2] Koizumi S., Watanabe K., Hasegawa M. & Kanda H. Ultraviolet Emission from a Diamond pnJunction. Science 292, 1899–1901 (2001). 11397942 10.1126/science.1060258

[b3] Watanabe K., Taniguchi T. & Kanda H. Direct-bandgap Properties and Evidence for Ultraviolet Lasing of Hexagonal Boron Nitride Single Crystal. Nat. Mater. 3, 404–409 (2004). 15156198 10.1038/nmat1134

[b4] Gu B. X., Xu C. X., Yang C. S., Liu Q. & Wang M. L. ZnO Quantum Dot Labeled Immunosensor for Carbohydrate Antigen 19-9. Biosens. Bioelectron. 26, 2720–2723 (2011). 20961745 10.1016/j.bios.2010.09.031

[b5] Yang C., Xu C. X. & Wang X. M. ZnO/Cu Nanocomposite: A Platform for Direct Electrochemistry of Enzymes and Biosensing Applications. Langmuir 28, 4580–4585 (2012). 22309190 10.1021/la2044202

[b6] Yang C., Xu C. X., Wang X. M. & Hu X. Quantum-dot-based Biosensor for Simultaneous Detection of Biomarker and Therapeutic Drug: First Steps toward An Assay for Quantitative Pharmacology. Analyst 137, 1205–1209 (2012). 22245799 10.1039/c2an15894a

[b7] Ren X. L. *et al.* White Light-Emitting Diode From Sb-Doped p-ZnONanowire Arrays/n-GaN Film. Adv. Funct. Mater. 25, 2182–2188 (2015).

[b8] Zhang X. H. *et al.* Bandgap engineering of GaxZn1–xO nanowire arrays for wavelength-tunable light-emitting diodes. Laser Photonics Rev. 8, 429–435 (2014).

[b9] Bagnall D. M. *et al.* Optically Pumped Lasing of ZnO at Room Temperature. Appl. Phys. Lett. 70, 2230–2232 (1997).

[b10] Lu J. F. *et al.* Plasmon-Enhanced Whispering Gallery Mode Lasing from Hexagonal Al/ZnO Microcavity. ACS Photonics 2, 73–77 (2015).

[b11] Chu S. *et al.* Electrically Pumped Waveguide Lasing from ZnO Nanowires, Nat. Nanotechnol. 6, 506–510 (2011). 21725304 10.1038/nnano.2011.97

[b12] Dai J., Xu C. X. & Sun X. W. ZnO-Microrod/p-GaN Heterostructured Whispering-Gallery-Mode Microlaser Diodes, Adv. Mater. 23, 4115–4119 (2011). 21812039 10.1002/adma.201102184

[b13] Zhu G. Y. *et al.* Ultraviolet Electroluminescence from Horizontal ZnO Microrods/GaN Heterojunction Light-emitting Diode Array. Appl. Phys. Lett. 101, 041110 (2012).

[b14] Dai J., Xu C. X., Zheng K., Lv C. G. & Cui Y. P. Whispering Gallery-mode Lasing in ZnO Microrods at Room Temperature. Appl. Phys. Lett. 95, 241110 (2009).

[b15] Zhu H. *et al.* Ultralow-Threshold Laser Realized in Zinc Oxide. Adv. Mater. 21, 1613–1617 (2009).

[b16] Zhang X. M., Lu M. Y., Zhang Y., Chen L. J. & Wang Z. L.Fabrication of a High-Brightness Blue-Light-Emitting Diode Using a ZnO-Nanowire Array Grown on p-GaNThin Film. Adv. Mater. 21, 2767–2770 (2009).

[b17] Sun X. W., Huang J. Z., Wang J. X. & Xu Z. A ZnO Nanorod Inorganic/Organic Heterostructure Light-Emitting Diode Emitting at 342 nm. Nano Lett. 8, 1219–1223 (2008). 18348540 10.1021/nl080340z

[b18] Lu J. F. *et al.* Improved UV Photoresponse of ZnO Nanorod Arrays by Resonant Coupling with Surface Plasmons of Al Nanoparticles. Nanoscale 7, 3396–3403 (2015). 25626917 10.1039/c4nr07114j

[b19] Lin Y. *et al.* Localized Surface Plasmon Resonance-Enhanced Two-Photon Excited Ultraviolet Emission of Au-Decorated ZnO Nanorod Arrays. Adv. Opt. Mater. 1, 940–945 (2013).

[b20] Li J. T. *et al.* Graphene Surface Plasmon Induced Optical Field Confinement andLasing Enhancement in ZnO Whispering-Gallery Microcavity. ACS Appl. Mater. Interfaces 6, 10469–10475 (2014). 24950411 10.1021/am502043f

[b21] Qiao Q. *et al.* Localized surface plasmon enhanced light-emitting devices. J. Mater. Chem. 22, 9481–9484 (2012).

[b22] Shen H. *et al.* Stable surface plasmon enhanced ZnO homojunctionlight-emitting devices. J. Mater. Chem. C 1, 234–237 (2013).

[b23] Okamoto K. *et al.* Surface-plasmon-enhanced Light Emitters Based on InGaN Quantum Wells. Nat. Mater. 3, 601–605 (2004). 15322535 10.1038/nmat1198

[b24] Cheng P. H., Li D. S., Yuan Z. Z., Chen P. L. & Yang D. R. Enhancement of ZnO Light Emission via Coupling with Localized Surface Plasmon of Ag Island Film. Appl. Phys. Lett. 92, 041119 (2008).

[b25] Cheng C. W. *et al.* Surface Plasmon Enhanced Band Edge Luminescence of ZnO Nanorods by Capping Au Nanoparticles. Appl. Phys. Lett. 96, 071107 (2010).

[b26] Niu B. J., Wu L. L., Tang W., Zhang X. T. & Meng Q. G. Enhancement of near-band edge emission of Au/ZnO composite nanobelts bysurface plasmon resonance. CrystEngComm 13, 3678–3681 (2011).

[b27] Kwon M. K. *et al.* Surface-Plasmon-Enhanced Light-Emitting Diodes. Adv. Mater. 20, 1253–1257 (2008).

[b28] Zhang S. G. *et al.* Localized surface plasmon-enhanced electroluminescence from ZnO-based heterojunction light-emitting diodes. Appl. Phys. Lett. 99, 181116 (2011).

[b29] Zhang S. G. *et al.* Optimization of electroluminescence from n-ZnO/AlN/p-GaN light-emitting diodes by tailoring Ag localized surface plasmon. J. Appl. Phys. 112, 013112 (2012).

[b30] Liu W. Z. *et al.* Localized surface plasmon-enhanced ultraviolet electroluminescence from n-ZnO/i-ZnO/p-GaN heterojunction light-emitting diodes via optimizing the thickness of MgO spacer layer, Appl. Phys. Lett. 101, 142101 (2012).

[b31] Liu W. Z. *et al.* Enhanced Ultraviolet Emission and Improved Spatial Distribution Uniformity of ZnO Nanorod Array Light-emitting Diodes via Ag Nanoparticles Decoration. Nanoscale 5, 8634–8639 (2013). 23897294 10.1039/c3nr02844e

[b32] Qiao Q. *et al.* Surface plasmon enhanced electrically pumped random lasers. Nanoscale 5, 513–517 (2013). 23203252 10.1039/c2nr32900j

[b33] West P. R. *et al.* Searching for Better Plasmonic Materials. Laser Photonics Rev. 4, 795–808 (2010).

[b34] Lu J. F. *et al.* Direct Resonant Coupling of Al Surface Plasmon for Ultraviolet Photoluminescence Enhancement of ZnO Microrods. ACS Appl. Mater. Interfaces 6, 18301–18305 (2014). 25244031 10.1021/am505492r

